# A Statewide Model for Assisting Nursing Home Residents to Transition Successfully to the Community

**DOI:** 10.3390/geriatrics3020018

**Published:** 2018-04-10

**Authors:** Darci Buttke, Valerie Cooke, Kathleen Abrahamson, Tetyana Shippee, Heather Davila, Rosalie Kane, Greg Arling

**Affiliations:** 1 Minnesota Board on Aging, Saint Paul, MN 55101, USA; darci.buttke@state.mn.us; 2 Valerie Cooke, Minnesota Department of Human Services, Saint Paul, MN 55101, USA; valerie.cooke@state.mn.us; 3 Kathleen Abrahamson, School of Nursing, Purdue University, West Lafayette, IN 47906, USA; garling@purdue.edu; 4 Tetyana Shippee, School of Public Health, University of Minnesota, Minneapolis, MN 55455, USA; tshippee@umn.edu (T.S.); wood0132@umn.edu (H.D.); kanex002@umn.edu (R.K.)

**Keywords:** caregiving–informal, decision making, evaluation

## Abstract

Minnesota’s Return to Community Initiative (RTCI) is a novel, statewide initiative to assist private paying nursing home residents to return to the community and to remain in that setting without converting to Medicaid. The objective of this manuscript is to describe in detail RTCI’s development and design, its key operational components, and characteristics of its clients and their care outcomes. Data on client characteristics and outcomes come from the Minimum Data Set, staff assessments of clients and caregivers, and Medicaid eligibility files. Most clients transitioned by the RTCI had entered the nursing facility from a hospital. Clients overwhelmingly wanted to return to the community and fit a health and functional profile making them good candidates for community discharge. Most clients went to a private residence, living alone or with a spouse; yet, adult children were the most frequent caregivers. At one year of follow-up 76% of individuals were alive and living in the community and only a small percentage (8.2%) had converted to Medicaid. The RTCI holds promise as a successful model for states to adopt in assisting individuals who are at risk to become long stay nursing home residents instead to return to the community.

## 1. Introduction

The public and private costs of long term care in the United States (US) will increase dramatically in the future, with nursing home and continuing care retirement facility costs projected to rise to $260 billion by 2026 [1]. Although most post-acute care in the US is publically financed through Medicare, financing for longer-stay nursing home residents, who incur the bulk of nursing home costs, is through the public Medicaid program and private out-of-pocket expenditures. Many individuals begin their nursing home stay paying privately, but then become Medicaid eligible when their assets are exhausted by paying for nursing home costs.

The first 90 days after admission is a critical period for making decisions about returning to the community, or remaining in the nursing home and becoming a long-stay resident [2]. The Federal Money Follows the Person (MFP), which many states have implemented to facilitate community discharge, is directed at Medicaid nursing home residents who have stays of greater than 90 days [3]. The number of MFP transitions has been small, e.g., 11,000/year, and only about one-third have been age 65 or older [4]. In contrast, 11–13% of mainly elderly residents who remained in nursing homes past 90 days have been determined to have relatively low care needs and could potentially be served more appropriately in a community setting [5,6].

Although Medicaid spending for long-term care has garnered much attention, the decision to become a permanent nursing home resident is of considerable importance for private paying residents who face large future out of pocket costs, and for the government because these individuals are at risk of exhausting their assets and converting to Medicaid.

Minnesota’s Return to Community Initiative (RTCI) is a novel, state-wide initiative to assist private paying nursing home residents to return to the community early in their stays, e.g., 60–90 days after admission. It complements Moving Home Minnesota (the state’s MFP program) and other programs aimed at Medicaid residents. The RTCI is administered by the Minnesota Board on Aging and it operates within the framework of the Older Americans Act and Area Agencies on Aging. The RTCI has been quite successful, winning awards for innovation, collaboration, and nursing home quality improvement from the Humphrey School of Public Policy at the University of Minnesota, Harvard Kennedy School of Government, Pioneer Institute, and SCAN Foundation.

In this manuscript, we will trace RTCI’s development, describe its key operational components, describe characteristics of its clients and their care outcomes, discuss challenges in implementing and evaluating RTCI, and present recommendations to other states and those in other countries that may be considering a similar initiative.

### 1.1. Development of the RTCI Model

The goals of the RTCI are to facilitate consumer choice in care setting and to achieve cost savings for the consumer and the Medicaid program. The RTCI’s focus on privately paying nursing home residents is unique nationally; most states have not delved into transitions for the privately paying nursing home residents. Like many states, Minnesota has a system of home and community services for Medicaid residents who need support in transitioning back to the community. Privately paying individuals, on the other hand, are often required to provide for their own discharge planning needs.

The potential for Medicaid savings was an important consideration in convincing state policy makers to undertake RTCI. Policy makers felt savings could be achieved by moving individuals from a nursing home to a potentially less costly community setting. Early community transition could delay or avoid Medicaid conversion.

The RTCI began in 2010 on a small scale with nine transitioned residents per month. It rose gradually to 42/month in 2013. The RTCI was expanded in 2014 with a base funding increase that has resulted in approximately 100 transitions per month. Currently there are 23 community living specialists, four case aides, two follow up specialists, all employed by regional Area Agencies on Aging. Two state units on aging staff support the initiative with technical assistance. The main program costs are for the 23 Community Living Specialists who are distributed across Area Agency on Aging regions and who are assigned nursing homes in their region.

The efficient use of resources was an important consideration in design of the RTCI. Rather than attempt to reach all private paying nursing admissions, many of whom would leave for the community on their own, the RTCI targeted a subset of residents early in their stays who were the best candidates for transition assistance and were at risk of remaining in the nursing home permanently. Residents meeting targeting criteria (described below) are contacted by the CLS staff to determine if they would like RTCI assistance leaving the facility. Other Non-Medicaid residents can also receive RTCI assistance if they are referred by the nursing facility or if they or family members seek help.

The RTCI’s potential for increasing community discharges for private paying residents was threatening to the nursing home industry because of potential loss of revenue. The RTCI came in the context of declining nursing home utilization, accompanied by a volatile nursing home market and unpredictable reimbursement rates. To allay the industry’s fears and to achieve provider buy-in, the RTCI staff undertook a concerted effort to engage the industry in support of the RTCI.

The RTCI staff strived for a high level of transparency and nursing home provider engagement. Program staff used business process modeling techniques to involve discharge planners and nursing home administrators in design of the RTCI. Three workgroups were created with internal and external stakeholders to design the resident intake and interview process; devise marketing strategies with nursing homes, trade associations and home and community based providers; and construct protocols for implementing the service. Program staff also adopted a comprehensive assessment and services planning process, accompanied by a set of service identification tools. The intake assessment, later named the Community Planning Tool, was field tested with willing nursing homes residents.

The RTCI staff was careful to manage the message with providers focusing in how helping lower acuity and less dependent individuals to move home opened up possibilities for an increase in higher need, higher reimbursement residents. A range of methods were used to market the RTCI. These included holding 10 community forums throughout Minnesota; preparing a booklet for use by consumers and family caregivers; sponsoring webinars; and making presentations at industry conferences.

### 1.2. Implementation of RTCI

The leadership of the RTCI staff has given considerable attention to the design of the RTCI, and has made continuous improvement as experience was gained through implementation. Of particular importance are staff qualifications, training procedures, and protocols.

Senior LinkAge Line^®^: The primary platform for the RTCI is Senior LinkAge Line^®^, which is the channel for the Aging and Disability Resource Center that serves people age 60 and older and is administered by the Minnesota Board on Aging. The Minnesota Board on Aging operates the Aging and Disability Resource Center as a networked model with many partners, which provides an ideal setting for the RTCI because of its statewide coverage and array of information and referral services open to all elderly and disabled individuals.

Staff Qualifications: Senior LinkAge Line^®^ Community Living Specialists, designated staff who provide the Return to Community initiative at the local level, hold a minimum of a bachelor’s degree from an accredited college or university in nursing, social work, gerontology or related human services field. The 23 current Community Living Specialists are Registered Nurses and Licensed Social Workers. Experience in care coordination and case management is essential. Previous employment in a nursing home is also a valuable asset for navigating the complex environment of long-term care.

Training Procedures: Community Living Specialists are employed by the Area Agencies on Aging and follow Area Agency on Aging internal training procedures with addition of training specific to the RTCI. Trainings include review of service provision protocols, documentation in the secure client tracking tool, use of secure chat and telephone systems, data privacy, and vulnerable adult mandated reporting. New Community Living Specialists shadow an experienced staff in their own region as well as in at least one other region. State program staff conduct a two-day site visit to observe protocol adherence once the Community Living Specialist has been employed for three months.

Monitoring and Performance Review: The RTCI has implemented a process for continuous quality improvement for the Community Living Specialists, including technical support through monthly conference calls and on an ad hoc basis as needed. State RTCI staff produces monthly regional and state level dashboards that are shared with the Community Living Specialists and program leaders. Staff level dashboards are provided on a quarterly basis to the Area Agency on Aging Directors, as well as Community Living Specialists and their supervisors. Dashboard metrics include: number of monthly discharges, compliance with obtaining written consent, data completion rates, and results from consumer satisfaction surveys. Community Living Specialists can run their own reports for personal self-assessment and record accuracy. These reports also show for each nursing home the size of its target list, number of RTCI-assisted transitions, and number of targeted residents still in the nursing home at 90 days. Community Living Specialists can use these metrics to reach out to nursing homes with low transition rates and high percentages of residents remaining in the facility.

Software Support: Secure, end to end encrypted software is used by the Senior LinkAge Line^®^ to document and store all consumer interactions as well as communicate with consumers, caregivers, and providers. Resource House Web-Based Referral, which is a secure client tracking tool and Revation Communicator, a secure chat and telephony system, are owned by Revation LinkLive™. These tools have had a third-party security review to ensure private data is secure.

Targeting: Resident target lists are produced weekly from residents’ nursing home Minimum Data Set admission assessments. The Minnesota Board on Aging downloads Minimum Data Set assessments from federal government servers and then prepares a resident file for use by the RTCI staff. Rapid turnaround of Minimum Data Set data facilitates timely targeting. Variables are examined to determine if the consumer meets the target profile. On average, the list contains 150 consumers each week. Consumers appear on the target list if they were not Medicaid eligible at admission and have:

- Resided in a nursing facility for at least 45 days;
- Expressed a desire to return to a community setting, based on a question in Section Q of the Minimum Data Set assessment; and
- Met the targeting threshold, i.e., 70 or more points on a 1–100 scale, based on their health and functional characteristics as recorded upon admission.

In-person Visits: The Community Living Specialists conduct in-person visits to each consumer on the target list. In-person visits are conducted to ensure consumers receive unbiased information regarding their options for residing in the community and make consumers aware of the right to live in the least restrictive environment. During the visit, the Community Living Specialist explains the free service that is available for discharging back to the community as well as the ability to receive five years of ongoing follow up. If the consumer agrees to the assistance, a release of information is obtained. The release gives the Community Living Specialist access to the medical chart, ability to speak with nursing facility staff and other health care providers, and collect private information for data analysis and evaluation. If the consumer does not consent to assistance from the RTCI, discharge tracking occurs via phone to ensure an outcome is documented for monitoring and evaluation purposes.

Transition Process: The RTCI transition process begins when the Community Living Specialist carries out an interview with the consumer and primary caregiver to determine the needs of the consumer both physically and mentally. The interview form, known as the Community Planning Tool, collects information on resident cognitive status, emotional status, function and well-being, and need for community services to supplement the Minimum Data Set assessment data. As a result of the interview, the Community Living Specialist may recommend the consumer apply for state assistance through Medicaid or a home and community-based services waiver. For veterans, consumers will be referred to the local County Veteran Service Officer to determine benefit eligibility. Lastly, if the consumer is facing barriers to discharge, a referral to the Ombudsman for Long-term Care may be necessary.

Care Planning: Consumer-centered plans, known as Community Living Support Plans, are developed based on the physical, social, and emotional needs of the individual as collected in the Community Planning Tool. Consumers receive service and provider options in order to understand the depth and breadth of the services available to them as well as any risks that they need to manage. Services are provided at a cost directly to the consumer or through a government service when available. A support plan is prepared with a summary of all the services for the consumer when they leave the facility. The plan contains information from the nursing home as well as community agencies, durable medical equipment suppliers, and even their primary care doctor. This single support plan document gives the consumer a comprehensive view of what to expect as they transition to a new setting. Not all individuals may be able to live in a home setting and, thus, other more structured options are explored such as adult foster care, group homes or assisted living.

Post-Discharge Visits: Once the consumer discharges, an in-person visit is conducted within 10 days with a phone call occurring within 72 h. If the consumer needs more immediate follow-up, the Community Living Specialist will visit within 72 h of discharge with a phone call occurring at 10 days post-discharge. This flexibility exists based on lessons learned since 2010 and providing person centered care based on the consumer and caregiver preference. During the in-person visit several action steps are taken by the Community Living Specialist to help the consumer transition back to a community setting:

- Verify consumers understanding of medications;
- Review emergency backup plan;
- Ensure prescribed medications are filled and available;
- Conduct medication reconciliation;
- Ensure primary care physician appointment is scheduled;
- Ensure services have arrived as applicable;
- Make addition caregiver and consumer referrals, if needed.

After the first two follow up sessions, ongoing follow up occurs at 30, 60, and 90 days post-discharge either by phone or in-person. If desired, consumers can receive follow-up for five years. During follow up sessions, information is collected including difficulties managing at home, recent hospital admissions or emergency department visits, and caregiver burden. With new information, RTCI staff can modify care plans to offer consumers and caregivers additional assistance, such as caregiver support groups, caregiver training, adult day care (for respite), home delivered meals, assistance with functional needs, or medication management devices.

## 2. Research Design and Methods

The RTCI has benefited from a comprehensive mixed methods evaluation with multiple objectives, including describing client and caregiver characteristics, tracking nursing home discharges and readmissions, estimating Medicaid conversion rates and expenditures, and evaluating transition processes. We report selected findings from that evaluation. In the early stages of RTCI planning, researchers from Purdue University and University of Minnesota had developed a targeting model for the RTCI. The Minnesota Board on Aging partnered with these researchers in the evaluation of the RTCI. The evaluation team obtained support through a Health Services Research Demonstration and Dissemination Grant (R18) from the Agency for Healthcare Research and Quality. The evaluation began in 2012 and continued through 2017. The evaluation focused on: RTCI’s outcomes—increasing resident transitions to the community, delaying Medicaid conversion, and achieving Medicaid savings; and RTCI processes—staff activities, nursing home engagement, and transitioned residents and family caregiver experiences. The Minnesota Board on Aging transmits RTCI program data periodically to the evaluation team, from which evaluators prepare quarterly reports on RTCI outcomes and processes that are shared with Area Agency on Aging Directors and Community Living Specialists.

In this manuscript, we describe the characteristics of individuals who met target group criteria and were transitioned by the RTCI program. We describe their health and functional status at admission, resident and informal caregiver characteristics at discharge, and outcomes at one year after transitioning. In addition, we compared targeted residents transitioned by RTCI to those who declined RTCI assistance and chose to remain in the nursing home past 90 days.

The primary data sources for resident characteristics were the Minimum Data Set Assessment, and the RTCI Community Planning Tool completed for the resident by the Community Living Specialist at the point of transition. Variables from the Minimum Data Set were: admitted from an acute care hospital, gender, marital status, age, and preference for community discharge. Use of a nursing home in the prior two years was taken from the historical data. Variables from the RTCI community planning tool were: incontinent of bowel or bladder, moderate/severe cognitive impairment, weekly behavior problems, dependency in bed mobility, transferring, eating, or toileting: (independent 1–4, moderate dependence 5–12, or severe dependence 13–16); high risk medications (psychotropic medication, anti-coagulant/platelet medication, analgesic medication, insulin/sulfonylureas medication, anticholinergic medication); fall risk (concern with falling, balance/vertigo concern, fell in nursing home); living arrangement at discharge (private residence with spouse/partner, private residence lives alone, assisted living, other); primary caregiver at discharge (adult child, spouse/partner, other, none). Other study variables were Medicaid conversion date from Medicaid eligibility files and date of death from Minnesota Vital Statistics records.

Data on 1216 RTCI transitioned residents and 926 of their caregivers, and 2044 targeted residents remaining in the nursing home at 90 days were taken from January–December 2016, the most recent 12 months for which we had complete data. In tracking 12-month outcomes, we drew data for a cohort of 925 RTCI transitioned residents who were admitted from April 2015 to March 2016. In order to obtain complete Medicaid eligibility data, we tracked Medicaid conversion for a cohort of 820 comparable admissions 15 months earlier, from January–December 2014.

## 3. Results

The RTCI program has assisted in the transition of 4305 residents from its inception through 2016. The number of resident transitions increased from 38/month in 2013 to 69/month in 2014. It has remained steadily at 90–100/month during 2015 and 2016. The target list has accounted for 58% of transitions, while 42% have come from a documented desire to return home from the Minimum Data Set section Q or facility referrals. The average length of stay at community discharge was 68 days. 47% were transitioned in <60 days, 31% from 60–90 days, and 22% in >90 days. Among residents targeted by the RTCI, half choose to remain in the nursing home, 14% received RTCI assistance, and 36% left the facility without RTCI assistance.

Table 1 presents characteristics of RTCI transitioned residents based on their status at initial admission to the facility as measured by the Minimum Data Set assessment. Their characteristics reflect the targeting profile which heavily weights variables indicative of the probability of community discharge. Nearly everyone (99%) had a preference for community discharge (Section Q1a), 93% were admitted to the facility from an acute care hospital, and 59% had no prior nursing home use in the prior two years. Although a majority were unmarried and female, substantial percentages were male (39%) and married (40%). Only about one-third of transitioned residents were age 85 or older. Only small percentages of residents had moderate to severe cognitive impairment (14%) or behavioral problems (7%); however, 37% of residents were incontinent at least daily and 91% were in the moderate range for functional dependency.

When comparing the admission profile for targeted residents transitioned by RTCI with those who remained in the nursing home at 90 days (Table 1), residents remaining were somewhat older, unmarried, and more likely to be cognitively impaired. Otherwise, those who remained in the nursing home did not differ significantly from residents who transitioned. Among residents remaining in the facility, there was only a small change in functional status between admission and 90 days. Residents who remained, experienced a small decline in cognitive status; however, they improved in independence with activities of daily living with a significant decline in the proportion moderately dependent (92% to 73%) and an increase in independent (5% to 25%).

Most RTCI transitioned residents went to live in a private residence, either alone (30%) or with a spouse (31%) and 22% went to assisted living (Table 2). Only 16% went to live with an adult child or had another living arrangement. Nonetheless, an adult child was the primary caregiver for 36% of the transitioned individuals (Table 2). The spouse was the primary caregiver for 31% of residents, the vast majority of whom were living with their spouse. Only 17% of individuals indicated that they had no primary caregiver.

Transitioned residents had significant risk for medication problems and falls when they returned to the community. A very high percentage (90%) of individuals were on high-risk medications upon discharge. These medications included a psychotropic medication (57%), anti-coagulant/platelet medication (56%), analgesic medication (43%), insulin/sulfonylureas medication (19%), and anticholinergic medication (12%). Over half of transitioned residents (52%) expressed a concern with falling, 48% expressed balance/vertigo concerns, and 15% reported having a fall in the nursing facility.

In 91% of cases where the spouse was the primary caregiver, he or she provided care at least daily (Table 3). This contrasts with adult child caregivers where only 37% provided care daily, 52% provided care at least weekly, and 11% provided care less than once per week.

Ninety-four percent of transitioned individuals received formally provided services upon discharge from the facility (Table 3). A large percentage (76%) received care from a nurse or home health aide. Smaller percentages had alarms or other technology (54%), in-home or home delivered meals (34%), other in-home services, e.g., personal care, homemaker or chore (36%), or transportation (22%). Most of these services were provided by a home health agency or Area Agency on Aging.

When asked about difficulties they expected to encounter in caregiving, 47% of primary caregivers anticipated no difficulties. Small percentages anticipated difficulties with job limitations (10%), their own poor health (6%), not enough time (5%), limited finances (4%), long distance caregiving (4%), or a burden on the rest of the family (3%) (Table 3).

Most individuals (76%) were alive and living in the community at one year after their initial transition from the nursing facility (Table 4). Fifteen percent had died. Thirty-four percent had experienced one or more readmission to the nursing facility but only 9% were back in a facility at one year. A small percentage of individuals had converted to Medicaid: 8.2% overall, 5.5% in the community and 2.7% for those who returned to the nursing home. In findings not reported in the table, we found no significant differences in outcomes for targeted residents transitioned by RTCI and those who left the facility without RTCI assistance. However, targeted residents remaining in the nursing facility had significantly higher rates of mortality (31%) and Medicaid conversion (24%) at one year.

## 4. Discussion and Implications

The RTCI is unique in its focus on assisting private paying nursing home residents to transition to the community at a point in their stays where they are at risk of becoming long-stay. The RTCI appears to be meeting many of its goals. By responding to residents seeking assistance the program has assisted a substantial number of residents, over 1200 per year, and their caregivers across Minnesota; three-quarters of transitioned residents were alive and remaining in the community and only 8% had converted to Medicaid at 12 months.

Judging from the health, functional, and caregiving profile of transitioned residents, the RTCI is assisting private pay residents who desired to return and could appropriately be cared for in the community rather than a nursing home. Targeted residents were largely post-acute admissions with no prior nursing home use; had no or only mild cognitive impairment; few evidenced behavioral problems; had moderate functional dependency, and only about one-third were incontinent. Although many individuals lived alone, nearly everyone had a primary caregiver. Spouses, when available, provided daily care; while most adult children provided care at least weekly. After a year, three-quarters of transitioned individuals were alive and living in the community and only a small percentage converted to Medicaid.

### 4.1. Challenges

Despite its successes, a sizable proportion of residents who could potentially benefit from the RTCI do not transition from the nursing home. Among targeted residents fitting the community discharge profile, nearly twice as many decline the RTCI offer of assistance and remain in the nursing home. Residents remaining in the facility expressed a preference for community discharge when initially admitted and they were very similar in health and functional status to those who were transitioned. The Community Living Specialists inquired about their reasons for remaining in the facility. The most common reasons expressed by residents were their concerns about access to health care and their personal safety. Small percentages of residents (<10%) indicated that lack of family caregiving or community services was a barrier to leaving. In performing their roles as both counselors and service planners, Community Living Specialists are challenged by the complexity of resident and family decisions about care settings.

An additional challenge is posed by the narrowness of the RTCI targeting profile. Even though RTCI assistance is open to all private paying residents regardless of their length of stay, the targeting profile gives priority to residents who are in the facility at 60–90 days, and who, based on health and functioning, are the best candidates for community discharge. The Community Living Specialists and nursing home discharge planners have noted that some residents may benefit from a timelier intervention earlier in their stays. Other residents not fitting the profiles may have significant health and functional needs, yet they have the motivation and social resources for a successful transition back to the community. The RTCI must remain flexible in responding to individual needs, while making efficient use of staff resources.

Finally, the RTCI is faced with the challenge of demonstrating its impact on nursing home use and expenditures. RTCI’s goal of cost savings, particularly for the Medicaid program, is difficult to evaluate because of uncertainty about its impact on nursing home use. RTCI was implemented statewide and has been open to all non-Medicaid residents who seek assistance.

### 4.2. Limitations

Absence of a control group and the fact that residents self-select to receive the RTCI intervention raises the possibility of selection bias. Thus, the ability of the evaluation to detect a causal effect on nursing use is limited. In addition, the RTCI was implemented in a state, Minnesota, which is noted for its generous Medicaid home and community based long term care services. This fact could affect generalizability to other settings. On the other hand, Minnesota is similar to other states in the paucity of public support for disabled older people not receiving Medicaid. Finally, we note that authors of the article have been involved in the development and implementation of the RTCI. Although close involvement provides insights into the program, it also could introduce bias.

### 4.3. Replication in Other Settings

States considering replication of an RTCI-like model should consider lessons learned from the RTCI experience. First and foremost, state agency leadership must garner support from policy makers, secure funding, obtain the authority to approach private paying residents, and implement other structural features for the program. It took considerable time and effort to develop the RTCI administrative infrastructure and processes described above.

State agency leadership needs to anticipate and proactively address the natural resistance from the provider community. The RTCI leadership adopted a strategic relational engagement model with the provider community. This included communicating in a transparent manner, regular check-ins with key change agents, garnering feedback and making a concerted effort to modify the RTCI approach based on stakeholder inputs. Transformative efforts should be meaningful and responsive in order to build the credibility of the model over time.

Even after a concerted effort at engagement, a substantial number of facilities may not be receptive to a RTCI-like program. In 2016, for example, one-third of facilities had none of their targeted residents transitioned by the RTCI. When we compared to facilities that were active participants (RTCI transitions of 15% or more of targeted residents) there were no significant differences in location, admission rates, case mix index or, most importantly, the proportion of residents expressing a desire to return to the community. An extra level of effort may be required to engage unresponsive facilities. The Community Living Specialists must develop good working relationships with the nursing home discharge planners and staff, presenting themselves as a resource and not a threat to the nursing home’s business.

The Older Americans Act network may be the logical entity to administer an RTCI-like program. Minnesota is fortunate to have a well-developed network that facilitated implementation of RTCI. The Senior LinkAge Line^®^ is a virtual statewide single point of entry for initial consumer contact, while the network of Area Agencies on Aging has locally accessible staff that can meet consumers in person with an almost immediate response. Referrals coming into a single web portal or toll-free line allows for monitoring of quality to ensure the intake is appropriately handled. The Senior LinkAge Line^®^ is capable of handling a high call volume that can adapt to the cadence and flow of facility and consumer contacts. It also has a high market penetration rate as measured in recent surveys of older Minnesotans.

A key component of RTCI has been post-transition support to maintain people in the community. Community Living Specialists focus particularly on the first few days after discharge from the NH when residents are most vulnerable for medication problems, risk of falling, or other problems as they adjust to a new care setting. Another important feature of RTCI is continued CLS monitoring of service needs, satisfaction with living situation, and caregiver arrangements. The relatively low nursing home readmission rates and mortality among transitioned residents may be attributable to effective follow-up.

## Figures and Tables

**Table 1:** Characteristics of target group residents transitioned by the Return to Community Initiative (RTCI) and residents remaining in the nursing home at 90 Days.

	RTCI Transitioned Residents: Admission Profile ^a^ (N = 1216)	Remain in Nursing Home: Admission Profile ^b^ (N = 2044)	Remain in Nursing Home: 90 Day Profile ^b^ (N = 2044)
Nursing Home Admit from Acute Care	93%	93%	
No Nursing Home Use in Prior 2 Years	59%	63%	
Female	61%	63%	
Unmarried	60%	65% *	
Age 85+	31%	45% *	
Preference for Discharge	99%	99%	
Incontinent	37%	41%	42%
Moderate/Severe Cog Imp	14%	21% *	25% **
Weekly Behavior Problems	7%	9%	7%
Activities of Daily Living (ADL): Independent (1–4)	6%	5%	25% **
ADL: Moderate Dependence (5–12)	91%	92%	73% **
ADL: Severe Dependence (13–16)	3%	3%	2%

^a^ Status at admission for RTCI transitioned residents who were admitted January–December 2016. ^b^ Status at admission and at 90 days for residents remaining in the nursing home at 90 days and admitted October 2015–December 2016. * *p* < 0.01 RTCI transitioned admission profile compared to remain in nursing home admission profile. ** *p* < 0.01 remain in nursing home admission profile compared to remain in nursing home at 90 days.

**Table 2:** Characteristics of transitioned residents at transition from the nursing home (N = 1216 transitions).

Characteristics	%
Fall risk	
Concern with falling	52%
Balance/vertigo concern	48%
Fell in NH	15%
Living arrangement at discharge	
Private residence with spouse/partner	31%
Private residence lives alone	30%
Assisted living	22%
Other	17%
Primary caregiver at discharge	
Adult child	36%
Spouse/partner	31%
Other	16%
None	17%

**Table 3:** Informal care and formal services at transition from the nursing facility. (N = 1216 transitioned residents and N = 920 primary caregivers).

Variable	%
Frequency of caregiving by primary caregiver	
Spouse/partner	
Daily	91%
At least once per week	7%
Less than once per week	3%
Adult child	
Daily	37%
At least once per week	52%
Less than once per week	11%
Other	
Daily	31%
At least once per week	57%
Less than once per week	13%
Difficulties in caregiving expected by primary caregivers	
No difficulties	47%
Job limitations	10%
Poor health	6%
Not enough time	5%
Limited finances	4%
Long distance caregiving	4%
Burden on the rest of my family	3%
Services received by transitioned residents at discharge from the nursing facility	
Home health nurse or aide	76%
Alarms or other technology	54%
Personal care, homemaker or other home services	34%
Meals	36%
Transportation	22%
Case management or referral	4%
Caregiver support	2%
Financial	2%
Respite	2%
Adult day services	1%
Hospice	1%
No services	6%

**Table 4:** Outcomes at 365 Days after transition from the nursing facility (N = 925 ^a^, 820 ^b^).

Outcome	%
Status at one year ^a^	
Alive and living in the community	75%
Died	16%
Living in the nursing facility	9%
Readmission to the nursing facility ^a^	
No readmissions	66%
One readmission	27%
Two or more readmissions	7%
Medicaid conversion ^b^	
Converted to Medicaid in community	5.5%
Converted to Medicaid after nursing facility readmission	2.7%

^a^ Resident admissions from April 2015 to March 2016. ^b^ Resident admissions from January–December 2014.
